# Current Developments in Digital Quantitative Volume Estimation for the Optimisation of Dietary Assessment

**DOI:** 10.3390/nu12041167

**Published:** 2020-04-22

**Authors:** Wesley Tay, Bhupinder Kaur, Rina Quek, Joseph Lim, Christiani Jeyakumar Henry

**Affiliations:** 1 Clinical Nutrition Research Centre, Singapore Institute for Clinical Sciences, Agency for Science, Technology and Research (A*STAR), Singapore 117599, Singapore; wesley_tay@sics.a-star.edu.sg (W.T.); bhupinder_kaur@sics.a-star.edu.sg (B.K.); rina_quek@sics.a-star.edu.sg (R.Q.); joseph_lim@sics.a-star.edu.sg (J.L.); 2 Department of Biochemistry, Yong Loo Lin School of Medicine, Singapore 117596, Singapore

**Keywords:** food volume estimation, deep learning, dietary assessment, public health, digital health, personalized nutrition

## Abstract

Obesity is a global health problem with wide-reaching economic and social implications. Nutrition surveillance systems are essential to understanding and addressing poor dietary practices. However, diets are incredibly diverse across populations and an accurate diagnosis of individualized nutritional issues is challenging. Current tools used in dietary assessment are cumbersome for users, and are only able to provide approximations of dietary information. Given the need for technological innovation, this paper reviews various novel digital methods for food volume estimation and explores the potential for adopting such technology in the Southeast Asian context. We discuss the current approaches to dietary assessment, as well as the potential opportunities that digital health can offer to the field. Recent advances in optics, computer vision and deep learning show promise in advancing the field of quantitative dietary assessment. The ease of access to the internet and the availability of smartphones with integrated cameras have expanded the toolsets available, and there is potential for automated food volume estimation to be developed and integrated as part of a digital dietary assessment tool. Such a tool may enable public health institutions to be able to gather an effective nutritional insight and combat the rising rates of obesity in the region.

## 1. Introduction

In recent decades, overweight and obesity has become a global health concern with significant economic and social implications [1,2,3,4,5,6]. It has also led to the proliferation of many metabolic and lifestyle diseases including Type 2 Diabetes Mellitus. The World Health Organization reported in 2014 that 1 in 3 adults were overweight, and that 1 in 10 were obese globally [1]. In 2017, direct costs linked to obesity and its associated diseases in Singapore alone, were estimated by the Asian Development Bank Institute to be USD 5.05 billion, or 37.18% of the country’s total healthcare costs [7]. 

This increasing prevalence of over-nutrition, especially in the Southeast Asian region, has been attributed in part to rapid urbanization in the last few decades [2,8,9,10,11,12,13,14,15]. Urbanization is associated with a combination of dysfunctional food systems, an adoption of Western diets, increased psychological stress and sedentary behaviors, leading to unhealthy environments that contribute to the development of chronic diseases [7,13,16]. In countries such as Vietnam and Laos, exposure to urban environments have been associated with a three-fold increase in obesity [15]. 

To improve population-level dietary practices and address these impending public health issues, several countries are working on developing healthy, functional nutrition systems [1,2,12,17,18]. However, gathering accurate information required to identify the various nutritional issues has been a challenge for government institutions, researchers and dietitians [19,20]. The diets of Southeast Asians are considerably complex due to diet variety, consumption of composite foods and communal eating practices [12,21,22,23]. Furthermore, the validity and accuracy of traditional methods such as diet records and recall-based tools have been highly disputed. Under-reporting rates as high as 41% have been evidenced in some studies, and there is a need for technological innovation to improve the accuracy of dietary assessment, on both an individual as well as a population level [24,25,26,27,28,29,30,31,32,33].

There is a consensus among the Southeast Asian countries to develop stronger nutrition surveillance systems to provide greater insight into the nutrition situation, and facilitate the implementation of nutrition-focused policies [17]. Adopting a patient-centered approach is crucial in diagnosing and addressing nutritional gaps. Furthermore, the widespread availability of smartphone devices, computer vision technology and improved digital connectivity has opened doors to more precise methods of dietary assessment [34,35,36,37,38]. 

The aim of this paper will be to explore the technical and cultural hurdles that contribute to the difficulty in assessing diets on both an individual as well as a population level in the Southeast Asian region. The paper articulates the scope of current dietary recording methods; reviews the potential of newly available digital methods of dietary assessment; and considers the viability of their application to the Southeast Asian demography.

A literature review was conducted to identify current image-based digital technologies that assisted with estimating food volume. Relevant original research articles and reviews published between January 2008 and January 2020 were identified and were included for discussion in this paper. Briefly, the following string of search terms were used in Pubmed and IEEE Xplore Digital Library, with no language or other restrictions: “((image based) OR (food photo) OR (deep learning) OR (food image) OR (food photo)) AND ((food portion estimation) OR (dietary assessment) OR (food volume estimation) OR (calorie estimation) OR (food intake measurement))”. The electronic search was supplemented by manual searches through the reference sections of selected publications, as well as with linked articles that were found to have cited these particular publications.

## 2. Complexity of the Southeast Asian Diet

The significant dietary diversity in Southeast Asian countries is largely attributed to the many ethnic and cultural food practices, as well as the degree of past and present foreign influence in the region [23,39,40]. Being strategically situated along a major maritime East–West trade route, most countries in Southeast Asia were subject to some form of colonial governance for significant periods in the last few centuries. These factors have molded the cooking styles, taste profiles, as well as the ingredients available to each Southeast Asian country [23]. Chinese immigrants brought along dishes such as noodles served in a broth, curried, or stir-fried with a variety of ingredients; many different types of dumplings and steamed buns; stir-fried, braised and steamed vegetable, fish and meat dishes that pair well with rice [23]. Influence from the Indian subcontinent contributed to foods such as coconut-milk based curries, flatbreads and a myriad of spiced biryanis [23]. European traders and colonial rule brought along with them bread and other bakery products, pate, salads, as well as many types of vegetables and herbs like cassava, tomatoes and papaya into the region [23]. Many of these influences were adopted and integrated with local produce and flavors, resulting in significant variation throughout Southeast Asia. In more recent times, the wave of rapid globalization has also brought in a whole new set of flavors through the introduction of fast food into the region [23,41,42,43].

All Southeast Asian countries are plural societies characterized by the presence of a dominant ethnic majority and an array of ethnic minorities [23]. Therefore, ethnicity, culture and even religion has a pronounced impact on the choice of foods, types of local ingredients used, structure of meals and patterns of eating behavior. There are further distinctions between urban populations and rural villagers; between the wealthy and the poor; and between the educated and the less educated, and these factors greatly affect access, as well as choice of foods [39,41]. This level of diversity can make it difficult for consumption patterns and behaviors to be accurately defined on a population level [23,41,44]. 

### 2.1. Meal Settings and Eating Practices

Meals in Southeast Asia are generally communal in nature and sharing of food from central platters is the typical practice [23]. A common type of meal that demonstrates this practice is the consumption of rice paired with a variety of dishes such as curries, braised meats, steamed vegetables and soups [22,23,39,42]. These dishes are often shared between guests at the table and individuals pick food from the central platters onto their own plates or bowls to be eaten with rice. Family meals for most Southeast Asian ethnicities consist of different dishes laid out on a table at the same time, to be picked from as preferred by the diners. At formal events and functions, these meals are also frequently served in a buffet line, allowing diners to pick their preferred choice of food from a variety of selections. At some functions, dishes are served sequentially one after another at the dinner table [23]. 

The street food culture of Southeast Asia also facilitates the busy urban life. It serves an array of foods that can be easily purchased and consumed on the go, taken back home or brought to the workplace for consumption [14,42,45]. This can range from full meals such as soup noodles or fried rice, to mid-meal snacks such as meat skewers, sandwiches, wraps and dumplings, as well as an assortment of bite-sized local *kuih* (traditional Southeast Asian pastries and cakes) [23,45]. 

The purchase of food from these vendors is becoming more commonplace in recent years [42,46]. Many Southeast Asian foods involve complex spice and herb pastes that are time-consuming to prepare and broths that can take hours to simmer [23,42]. In the past, most of these meals were prepared at home but the increasing demands of the workforce have resulted in families having less time to make these authentic foods [45]. Increasingly, many urban families are choosing to either eat out at restaurants or food centers or to buy foods from street vendors to consume with rice at home [23,45]. In 2010, 60.1% of the general adult population in Singapore were found to eat out at least four times a week, and a 1990 survey in Bangkok showed that families spent about half their monthly food expenditure on foods prepared outside the household [23,42]. An increasing reliance on external food sources is a trend seen in many urban societies, and other rapidly urbanizing countries in Southeast Asia may also experience similar trends [14]. 

From a nutrition standpoint, the vast variety of food sources can make it challenging for individuals to have an in-depth understanding into the types and proportions of ingredients used. Aside from the different recipes used by chefs and establishments, portion sizes can also vary significantly between sources. This dynamic food landscape can make it difficult to define, much less identify, a standardized portion size and can complicate the matching of a food to an item or items in a nutrition table.

### 2.2. Personalization of Meals 

Many Southeast Asian foods involve a level of personalization. For example, noodle vendors in Malaysia or Singapore would ask customers for their preferred noodle type; if they prefer their noodles to be served dry or in a broth, and if they have a preference for chili or other sauces. Noodles from other Southeast Asian regions such as Vietnam and Thailand have similar features: Vietnamese rice noodles, also known as *Pho,* have platters of vegetables, herbs and condiments placed on the table for customers to help themselves and Thai boat noodles often have a tray of various dry and wet condiments placed at the table for diners to tailor their meals to their own personal tastes [23].

Economy rice is a type of dish consumed by many of the major ethnicities in Southeast Asia, and there are a variety of terms used to describe the meal such as *Mixed Vegetable Rice*, *Cai Fan*, *Nasi Campur*, *Nasi Padang* or *Banana Leaf Rice.* The meal typically involves the selection of 2 to 4 dishes from an array of 10 to 30 dishes to be eaten with rice [23]. Serving sizes for each of the dishes can be highly variable depending on the generosity of the stall attendant. Customers can also choose to have their rice doused with various types of curries or sauces, changing the overall profile of the meal to their liking [23]. This degree of customization is also prevalent in the many communal meals that Southeast Asians partake in. Dishes are scooped from shared platters and placed into an individual’s own bowl or plate, to be eaten in the order and quantity of their choosing [23,44]. 

Personalization and self-regulation is an integral part of Southeast Asian cuisine and can make assessment of population-wide consumption patterns problematic [40]. This can confound generalized assumptions and mask specific nutritional issues that may be faced by certain population groups, making it difficult for population-wide nutrition policies to be implemented effectively. 

## 3. Limitations of Current Dietary Recording Methods 

Diet recording is a prospective method of measuring dietary intake that requires respondents to log intake information over a period of time. It is also used extensively in many aspects of nutrition assessment [28,33]. This can come in the form of food diaries, weighted food records, or duplicate portion measures. This is unlike other tools such as the Food Frequency Questionnaires (FFQ) or the 24-hour diet recall, which are tools that rely on obtaining retrospective information about foods consumed in the recent past [28,33]. 

Diet records are considered to be more detailed than recall-based methods and are preferred as a means of dietary assessment for various reasons [24]. They reduce the degree of recall bias by not being dependent on the subject’s ability to remember consumed foods, as well as the interviewer bias given the need for detailed probing with recall-based methods [33]. Respondents are also trained before they commence the diet recording, and can thus be made aware of the necessary details to pay attention to. However, there are several limitations to the use of dietary records. 

Dietary records require respondents to record precise amounts of food, often over a few days, and sometimes over weeks [24,32,47,48,49]. This places a huge burden on respondents to ensure that the information is accurate and precise. Respondents are required to be highly motivated and to commit the time and effort needed to complete lengthy records. As a result, high rates of dropout have been evident after three consecutive days of recording [27,33]. 

In addition to being tedious, the inherent act of recording one’s diet has been known to change a person’s dietary practices, and research has shown shifts towards healthier foods and reduced energy intake in participants after three consecutive days of recording [24,33,47,50]. To reduce the amount of effort required, respondents have also been noted to progressively record less detail of their meals over the duration of the recording period, sometimes omitting more complex items, and even changing their diets to favor simpler foods [27,32,47,48,49]. Increased dietary awareness as a result of pre-study participant training may also affect a person’s dietary practices and present an inaccurate assessment of their habitual consumption patterns. The fear of being criticized for poor eating habits may also lead respondents to omit less desirable foods and/or over-quantify healthier foods. This is known as social desirability bias and can affect the integrity of the record [24,28,32].

Poor literacy and education levels can compound a lack of clarity and attention to portion sizes and ingredients used, in turn affecting the accuracy of the dietary records [24,27,32,51,52,53]. Despite the fact that most prospective studies begin with a degree of training for respondents, the effectiveness and receptiveness of the training is dependent on the participants’ abilities to grasp and execute these concepts [24,32,51,52]. This is further amplified by the complexity of Southeast Asian diets, and the reliance on food consumed outside the home can make it difficult for individuals to be aware of all the ingredients in the food [32,44]. Mixed dishes are featured heavily in Southeast Asian cuisine and are known to be especially difficult to quantify given the different proportions of ingredients used in different settings [54]. The shared or multi-course meals common in Asian demographics may also further complicate the quantification of consumed foods, and respondents may leave out many details when attempting to record their intake [44].

Significant errors can also occur at the point of coding, especially in the case of open-ended diet records [33,51,52,53,54,55]. These errors include: misinterpretation of portion sizes; unclear or inconsistent food names, particularly in the case of ethnic foods; or lack of detail with regards to food preparation methods and specific ingredients [54]. All of these factors require a degree of professional judgement on the part of the coder when the food records are interpreted, and this can differ between nutrition professionals [54]. Guan et al. found a 26% discrepancy rate when food record entries were verified against their respective FoodWorks analyses entered by coders, indicating that a certain degree of subjective extrapolation was required to match paper records with information available in the database [54]. The choice of analysis software and nutrient database can also add another layer of complication, with different tools having different values for similar items. As a result of these many factors influencing the outcome of dietary assessments, differences in total calories extrapolated from food records may be evident between dietitians or researchers within a team.

As mentioned earlier, dietary records require large amounts of resources per respondent, and are difficult to implement in large scale research, or public health settings [28,51]. Despite 7-day Diet Records being considered one of the better-regarded methods for dietary assessment, the number of participants that researchers are able to recruit is limited not only because of the difficulties faced by respondents, but due to the time-consuming nature of participant training and data processing [33,47]. At the end of studies, researchers may be presented with huge amounts of data to transcribe, and depending on the dietary complexity of the target group and the length of the study, this can put a significant strain on resources [54,56].

## 4. Digitization of Dietary Collection Methods

### 4.1. Feasibility of Going Digital

Advances in technology have alleviated some of the shortcomings of traditional dietary recording methods. Access to the internet, the availability of mobile phones, and the integration of cameras into the general population have expanded the toolset for dietary assessment [57,58,59,60]. 

Southeast Asia has shown a rapid growth in the use of technology and is a potential market for mobile healthcare apps, with internet usage in the region increasing from 12% in 2007 to 44% in 2017, mobile broadband subscriptions increasing from 1.3% in 2009, to 85% in 2017 [34,61]. These healthcare apps may require high initial costs to develop, but can greatly reduce the effort and uncertainty required for the collection and handling of data. This alleviates some of the burden of both respondents and researchers alike and improves the feasibility of dietary records in larger populations [24,51,52,61]. 

Currently, there are a multitude of digital dietary recording apps available [50]. The earliest forms began with simple text-based platforms that allowed users to type in the names and quantities of consumed foods [53]. Improvements to online communication platforms and smartphones have allowed apps to evolve beyond just text-based systems to include integrating image-based dietary recording, health coaching and dietary consultation into the functionality of the apps [38,62,63,64]. Further advances in computer vision and smartphone on-board processing capacity have also recently opened the door to image recognition and segmentation capabilities, indicating a potential for foods to be automatically identified and categorized as required for further processing [65]. 

### 4.2. Benefits of Digital Healthcare Solutions

One of the primary drivers for the use of digital solutions is its ability to personalize and individualize healthcare. This idea of personalized nutrition involves shifting the delivery of nutrition and lifestyle recommendations from a population level to an individual level, enabling greater adherence to nutrition goals and more effective behavior changes for users [58,59,66]. Digital applications enable healthcare professionals, researchers and even peers, to be in contact with subjects more frequently and for longer periods of time. This leads to more representative data of a person’s long-term diet coupled with an increased level of engagement, social support and feedback, allowing the delivery of more successful interventions [59,67,68]. By being more integrated in the lives of consumers, healthcare apps can assist with the translation of health education into application. Apps can provide nutrition education and allow for the setting of dietary goals. Many of these apps have been well received by consumers and facilitate improvements to the predictors of behavior change such as knowledge, self-efficacy and motivation [37,60,67,68,69,70,71,72]. Being able to be in contact with a healthcare professional also provides a layer of support whenever required, and this has been shown to improve engagement and satisfaction with the lifestyle change [38,58,60,62,63]. 

Using digital solutions also reduces the burden placed on respondents and healthcare professionals [53,60,67,73]. Many studies have found that users prefer the use of digital dietary recording over traditional paper methods for ergonomic and practical reasons [27,28,37,38,52,58,60,62,67,71,72,74]. Smart devices are highly integrated into modern lifestyles and are less intrusive to daily routines as users do not need to carry around an additional item such as a journal to record their diets with. In addition, these applications can also actively remind users to input their meal entries when required [50,70,75]. The ability to easily take photos of food also reduces the amount of textual input required by users. This makes the process less subjective and reduces the reliance on the respondents’ ability to recall or describe [24,28,32,33,63]. The simpler process requires less time and effort on the part of respondents, and therefore is less likely to impact research-related behavior changes that are often associated with tedious dietary records [24,27,32,47,48,76]. From a healthcare standpoint, variability of interpreted dietary results implicated during the coding process may also be substantially reduced with the automation of this process, potentially minimizing the chance for human error and standardizing the task of data extrapolation [55]. 

Organizationally, digital touchpoints are also expected to significantly reduce healthcare costs [33,56]. By enabling automated data capture and processing, the time required for the interpretation of diet records can be mitigated, decreasing the administrative load on healthcare professionals and researchers [33,55,56]. Patients can also be assessed and followed up remotely, reducing patient traffic in crowded healthcare institutions and reducing the geographical limitations faced by travelling clinicians in rural areas [53,56,73].

The level of personalized engagement and resource magnification that digital nutrition applications have the potential to provide is unprecedented. Adoption of these new forms of data collection could allow for nutrition data to be gathered in more detail, across larger populations, blurring the lines between individual- and population-level assessment methods. Given the increasing integration of digital platforms and devices into all walks of life, policy makers, researchers and dietitians should consider these avenues as a means of facilitating better health outcomes.

### 4.3. Limitations of Digital Healthcare Solutions

That being said, care must be taken to ensure successful and responsible adoption of digital solutions. Given that many apps have not been validated and may base recommendations and information on incorrect nutrient databases, both consumers and health professionals need to be wary of the accuracy of mobile applications [71]. Braz et al. assessed 16 apps in Brazil and found that despite most of them receiving high favorability from consumers (81.25% of apps receiving four stars and above), the energy values provided in many of the apps deviated by an average of 34% when compared to an officially regulated nutrition database, with discrepancies as high as 57% [71]. Apps that allow for new database entries to be added by consumers may be subjected to an additional layer of inconsistency. In one particular study, there was significant variation in caloric data between English-speaking and Portuguese-speaking users of the nutrition app *MyFitnessPal* because Portuguese entries were created by users and not by the company [70]. For these technologies to be accurate, these databases need to be regulated be professional health bodies that can provide credible and authoritative sources of evidence-based health and nutrition information.

The lack of regulation for health and lifestyle apps may be a concern with regards to the quality and efficacy of future apps. Issom et al. reviewed diabetes-focused mobile apps between 2010 and 2015, and found that only 8% of 53 publicly available apps were Food and Drug Administration (FDA)-approved; 4% were approved under the Health Insurance Portability and Accountability Act (HIPAA); and 6% obtained a CE marking which indicates conformity with health, safety and environmental protection standards [68]. Apps that focus solely on diet and diet recommendations are not considered mobile medical applications by the FDA and thus do not need to abide by their quality-control guidelines [77].

There are also some respondent burdens that may not be as easily resolved with the use of technology. Diet recording apps do not fully overcome methodological biases that traditional methods have with self-reporting [24,28]. Individuals have been shown to still have the tendency to under-report intake data, omit certain food choices to avoid social judgement or alter their diets when they are aware of an upcoming survey [78]. These issues could confound results in both individual- as well as population-level studies into nutrition and may be even harder to detect remotely in the absence of face-to-face interaction.

In the interest of data accuracy, current commercially available apps are still unable to determine absolute food volume and by extension, nutrient content of foods. Instead, these applications rely on user input or selection from a list of common serving sizes of identified foods for use in deriving estimations of nutritional values [65]. As discussed above, users may not be able to accurately quantify food and serving sizes have been shown to vary considerably even for similar foods [46]. To eliminate these discrepancies and reduce the variance associated with the quantification of food intake, an automated tool for food volume determination will eventually be needed to advance the field [28,51,79,80]. 

## 5. Recent Developments in Food Volume Estimation

**Figure 1:**
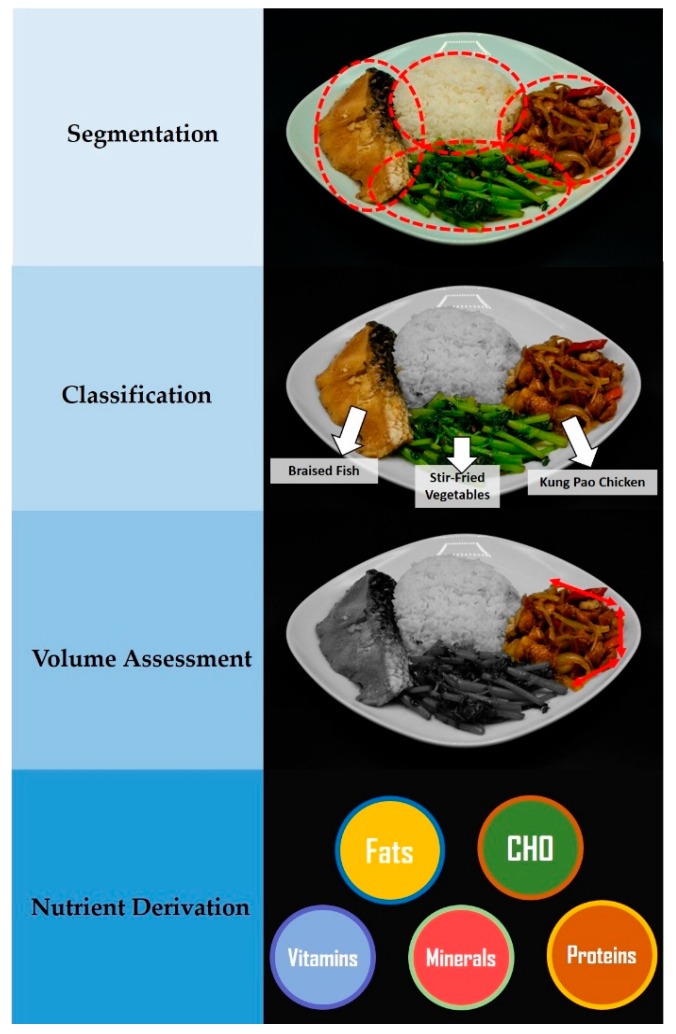
An illustration of the various steps of image-based automated dietary assessment—Segmentation, Classification, Volume Assessment and Nutrient Derivation.

In the field of automated vision-based dietary assessment, it has been established that images need to undergo a few steps of analysis: segmentation, classification, volume assessment and nutrient derivation (Figure 1) [81]. Segmentation uses computer-vision tools such as *Grubcut* to define the borders and separate the respective foods in the image; classification uses deep learning principles such as Convolutional Neural Networks (CNN) to identify the foods; volume assessment involves the determination of the volume of the identified segmented foods; and lastly, nutrient derivation matches the assessed volume with density and nutrient datasets to calculate the nutrients or calories contained within the foods in the image.

At present, the steps that involve segmentation, classification and nutrient derivation are well developed with well-prescribed methods that can perform the task well, subject to the integrity and precision of their respective training datasets [35,65,81,82,83,84]. However, the current state of the art of food volume estimation is not deployable into commercial apps due to many gaps and technological issues. With the developments in optics, computer vision and deep learning, this research area has seen many improvements that could hasten its eventual integration with the other aspects of dietary assessment. Some of the newer technologies have been summarized in Supplementary Table S1 and we will be reviewing them below.

### 5.1. Scale Calibration Principles

Volume estimation requires for scale references to be established within an image such that dimensional coordinates of the target object can be determined. Two-dimensional images are a collection of pixels and do not provide any indication of the relative sizes of the objects pictured in the image. Researchers have explored various methods of scale calibration, but they can be broadly categorized into whether a system requires an additional physical reference marker, also known as a fiducial marker, or if they are able to extrapolate scale via other digital means [85]. 

#### 5.1.1. Physical Fiducial Markers

Physical fiducial markers can come in many forms and serve as a reference point for dimensional calculations. The earlier variants were specialized equipment distributed by the researchers: a study by Yue et al. used standard plates of known diameters so that all food could be estimated relative to the size of the plate [86]; Jia et al. explored the positioning of a placemat outlined with special markings placed underneath the food [87]; and many studies utilized various forms of colored and/or chequered cards of known size placed next to food items to serve as a reference for scale calibration [75,88,89,90,91,92,93,94,95,96]. These methods can be useful in controlled environments such as in hospitals, schools or canteens, where the fiducial markers can be printed on trays, or standardized crockery can be used to provide a convenient degree of scale calibration within the pictures. However, for participants who are required to use these methods in free living conditions, being required to carry around an additional item may be inconvenient and may lead to poor compliance with study protocol [85].

Researchers have also experimented with using foods or tableware present in the picture as a basis for scale calibration. Based on the assumptions that Japanese rice grains are similar in size, and that most Japanese meals are frequently consumed with a bowl of rice present, Ege et al. proposed an ingenious system that utilized the grains of rice in the photo as the markers for volume estimation [97]. Though relatively accurate, with 84% of estimated results incurring less than 10% relative error, the method can be quite restrictive and culturally specific. This will unfortunately not work in other contexts where different types of rice are consumed, or when rice is not present in the meal. Akpa et al. also explored the use of another culturally-specific fiducial marker in the form of chopsticks [98]. By placing one chopstick on top of the bowl and the other on the table, they were able to deduce the estimated depth of the bowl from the differences in perceived length between the two chopsticks, obtaining promising results with a low relative error of 7%. These technologies could have much potential if their capabilities are further expanded to integrate multiple types of commonly available cross-cultural foods or tableware as markers. 

In the interest of ergonomics and usability, common everyday items of a known height such as a one-yuan coin and a credit card, have been trialed [91,99]. More personalized systems have enabled the use of a user’s thumb size to be calibrated for use as a fiducial marker in inferring scale. Users are then instructed to place their thumb next to their food when capturing images, allowing for a convenient marker unique to every individual user [100,101].

#### 5.1.2. Digital Scale Calibration

Given that the above methods require an additional step on the part of users and can affect compliance and adherence to a tool, researchers have experimented with alternative methods that circumvent the need for a physical fiducial marker. Some of these new tools involve the use of modern computer vision technology. This includes Stereo Vision, Structured Light, Augmented Reality and Depth Sensing.

Subhi et al. explored the use of eyewear outfitted with twin cameras for stereo imaging [102]. As the cameras were positioned at a pre-determined distance apart from each other, object dimensions could be calculated based off the positions of key features identified in the pair of images [102]. Shang and Makhsous et al. utilized a smart phone attachment that projected a structured light system consisting of multiple laser dots onto targeted foods [103,104]. This created a visible set of coordinates on the food from which distance and the food’s 3D model could be determined. Similarly, an extension of experiments by Jia et al. with the colored plate marker was one with the use of an elliptical ring projected by an LED spotlight [86]. These tools, though circumventing the need for a physical marker, required specialized equipment to be carried around and set up for the scale calibration to work. Hence, they may be inconvenient for users if used in free-living conditions. 

Tanno et al. and Yang et al. explored the use of Augmented Reality (AR) to enable volume estimation without the need for a physical fiducial marker [105,106]. In a study by Yang et al., the phone was placed on a flat surface, which allowed for a “table cloth” with grid markers to be projected into virtual space to serve as reference points for volume estimations [106]. Tanno et al. acquired the 3-dimensional coordinates of the world via the Apple ARKit framework, allowing for relative distance of reference points on the food object to be determined and quantified [105]. 

The increasing availability and accuracy of depth sensing technology in recent years has enabled the ability to detect form and shape and to establish the scale of objects beyond the limits of red/green/blue (RGB) images. Depth cameras such as the Microsoft Kinect or Intel RealSense have been used in fields of engineering and medicine, and are now being integrated into the field of nutrition [107,108,109,110]. Modern smartphones are also being outfitted with multiple cameras that also allow for stereo vision and depth perception. Given the ability to detect distance accurately, these modern methods using depth perception have enabled researchers to circumvent the need for a fiducial marker entirely [107,108,109,111].

### 5.2. Volume Mapping

Once the scale of the objects within the images has been determined, geometrical landmarks on the food items can then be established. This serves as the basis for volume to be extrapolated. 

#### 5.2.1. Pixel Density

**Figure 2:**
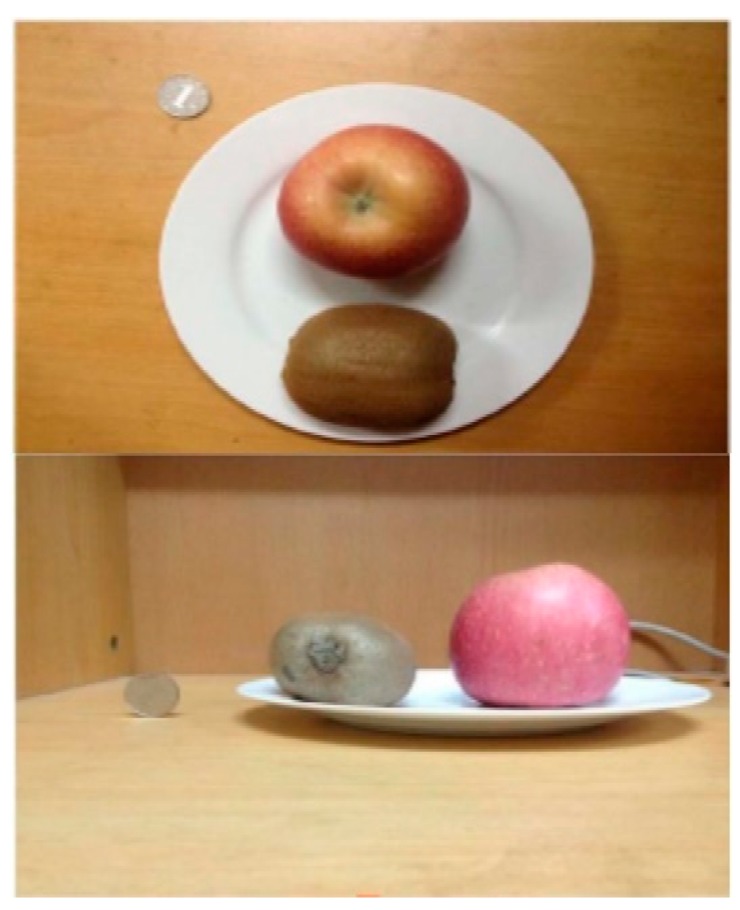
Measuring relative volumes by pixel density, Liang and Li. 2019.

As shown in Figure 2, Liang and Li utilized a method of volume estimation by determining the pixel of a One-Yuan coin and compared it to the amount of pixels taken up by the target food [99]. Geometric measurements were based off images taken from the top and side angles. The volume was then calculated with the use of one of three formulas, depending on whether the food was ellipsoidal, columnar, or irregular.

Zhang et al. performed volume estimation through a processing of captured 2D images by calculating the number of pixels per segment of food [112]. Though the authors commented that the counting of pixels led to a “reasonably good estimate”, this was not quantified in the paper. Okamoto et al. employed a similar method which compared the number of pixels occupied by foods in a 2D image to a reference object. Calories were then directly calculated with quadratic equations established with training data and achieved a relative error of 9%–35% for the three test foods [113].

#### 5.2.2. Geometric Modelling

Geometric modelling is the use of geometric shapes of known or easily calculable volumes such as prisms, ellipsoids, cylinders and rectangles. These shapes are projected and fitted onto identified, segmented foods in the presented image, allowing for food volume to be estimated. 

Subhi et al.’s eyewear-mounted stereo cameras allowed corners of food to be identified with the use of an edge-detection algorithm. This allowed a boundary cube to be defined by the detected points and projected into 3D space [102]. This method, however, resulted in empty spaces being factored in, leading to overestimations of volume, especially in the case of irregularly shaped objects. Volume derived from this geometric model proved to be a relatively accurate option, achieving a relative error of 2% to 13% [102]. 

**Figure 3:**
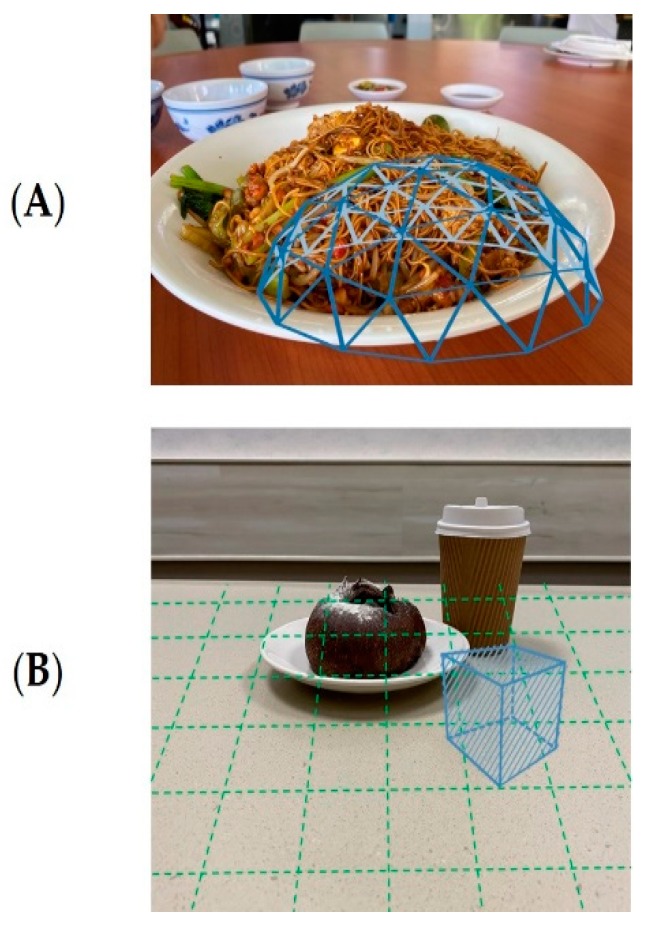
Food volume estimation using geometric modelling. (**A**) Movable spherical cap as adapted from Jia et al. 2014; (**B**) Projected variable cube using AR technology as adapted from Yang et al. 2019.

Researchers have utilized both user- and computer-assisted methods to map geometric models resembling food objects to serve as a basis of volume estimation. Woo et al. experimented with the use of prismatic and spherical models; Chae et al. experimented with the use of cylindrical and solid, flat-top models; and Jia et al. used a series of different shapes, including a spherical cap that could mimic the volumetric outline of foods piled on a plate (Figure 3A) [75,90,114]. These shapes were projected over foods and used as a basis for calculation of objects such as oranges (spherical), cups of beverages (cylindrical) and scrambled eggs (prismatic).

With the virtual tablecloth projected by their AR technology, Yang et al. allowed users to align a cube of known volume next to target foods for comparison (Figure 3B) [106]. This cube was able to be scaled to various volumes and allowed users to manually estimate the volume of the food against the volume of the cube. However, this study only provided a cube as a reference shape and hence, smaller, irregularly shaped foods were more challenging to measure.

With the aid of Apple’s ARKit framework, Tanno et al.’s proposed method allows users to use iPhone devices to project points into virtual space [105]. This allowed users to identify corners of foods, where a boundary box could be drawn. Quadratic equations were then applied to derive calories from the calculated boundary box. This method was innovative but restrictive given that the boundary box and quadratic equations used may also encounter problems with irregularly shaped foods.

#### 5.2.3. Machine and Deep Learning 

The use of deep learning principles has been experimented with in the field of volume estimation, primarily involving the use of network architecture such as Convolution Neuro Networks (CNN). CNN is a system that identifies and recognizes similarities in images, and can be used to amalgamate key volume, caloric and classification information of various types of foods [115]. This then allows these metrics to be predicted when new food images are supplied. 

Though not specifically targeting food volume, Ege et al. proposed a similar system that directly estimates calories based on ingredients and cooking directions found on various recipe websites [116]. The researchers created a calorie-annotated food image database that calculated calories from the ingredients listed in the recipe cards, and corresponded this information with the food images provided in the recipe listing. The relative calories of newly input foods are then estimated based on their similarities with the food images found in the database.

Isaksen et al. attempted a model that utilized machine learning for the entire assessment process of segmentation, classification, weight extrapolation and calorie derivation [117]. The training dataset consisted of images taken from the FoodX dataset created by researchers at the University of Agder. These training images were annotated with weights and nutrient values, and a ruler was placed beside photographed foods for scale. Though the system was able to successfully achieve every step of the process, it was inaccurate and incurred an average error of 62% [117]. 

Chokr M et al. experimented with a similar system for fast foods [118]. However, instead of obtaining caloric and volumetric information from ingredients listed in recipes, a dataset of 1000 images of six different categories of food were sampled from the Pittsburgh fast-food image dataset. The respective sizes and calories were annotated on the images with information obtained from online restaurant websites or nutrition databases. These images were then used for the training of the system. This system achieved a mean absolute error of only 9% with test images taken from the same dataset. However, the dataset was taken from a collection of 11 popular fast-food restaurants and the food contained a look that was largely similar to each other and may not have presented the same degree of a challenge as if the photos were taken in free living conditions [119]. 

#### 5.2.4. Depth Mapping

Depth mapping is a representation of the area occupied by an object in 3D space through a projection of voxels (volume pixels) [110]. This technology presents opportunities in terms of food volume measurement given the precise amount of detail that can be captured on irregularly shaped objects. A depth map of an object can be determined by various methods such as stereoscopic vision, structured light, depth sensing or deep learning methods. As mentioned earlier, these optical approaches are able to determine the relative distance of objects within the picture allowing for the 3-dimensional surface area of food to be calculated in pictures without the need for a fiducial marker [108,111]. The height is defined based on distance from the relative plane to the identified Y-axis coordinates and the volume can be calculated respectively.

**Figure 4:**
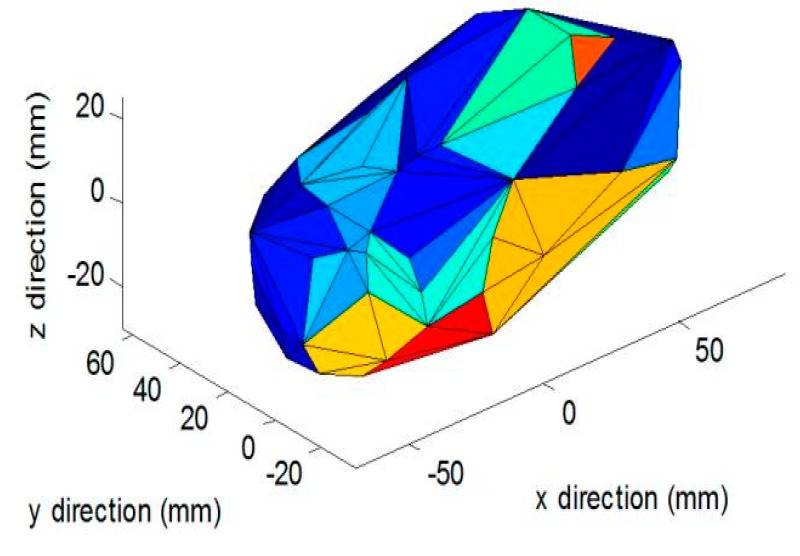
Depth map of a mango captured with a structured light system, Makhsous et al. 2019.

Dehais et al. utilized the technique of pixel matching with a pair of stereo images taken from above food to extrapolate vertical depth data with the aid of a chequered fiducial marker [89]. Matching of the positional variations of geometric landmarks between the two images allow for a voxel depth map to be constructed, and consequently, food volume can be determined (Figure 4). This method was relatively accurate, with a mean absolute percentage error (MAPE) between 7% to 10% on tested foods [89]. 

Makhsous et al. proposed the use of a mobile phone outfitted with a structured light attachment for food volume analysis [104]. This structured light system projected a series of dots onto food. Based on the distortions caused by the structures of the food, a 3D depth map can be plotted. Though the method had a low overall percentage error of 11%, it was not ergonomic for users. It required that users carry around an additional attachment for their smart phones, as well as taking a full 360-degree video of food that they would like to assess [104]. 

Ando et al.’s method utilized the multiple cameras on the iPhone X to produce depth images [111]. With the RGB-Depth (RGB-D) images captured by the device, he was able to collect information on the 3-dimensional structures of food allowing for more accurate volume assessment compared to other forms of 2D image analysis. The relative error ranged between 1% and 7% for the three types of food items that were calculated [111]. However, there are some limitations to this method in that it only takes into account the top surface of the food, given that any food area beneath the visible surface is occluded from view. All volumes below the visible surface of the food, to the reference plane, will therefore be included in its calculations by the system [111]. This may present a degree of noticeable inaccuracy in volume calculations in cases where food is positioned on sloped surfaces such as in a bowl, items that are overlapping each other, or in cases of irregularly shaped foods such as chicken wings.

Evidently, reducing the number of images required to be taken by users would be ergonomically ideal, but this can lead to inaccuracies in quantifying volume due to occlusions that are not visible in a single-view image [108,109,110,120]. To circumvent this issue, researchers have extended the use of depth sensing technology to integrate the aspect of deep learning, allowing 3D depth maps of food objects to be predicted based on visible surfaces [108,109,110,120]. This model works on the assumption that sufficient images and training will allow the system to understand the context of the scene, allowing for camera viewing angles, depth value of food objects, and occluded regions to be extrapolated from the images [108,109,110,120]. Unlike previous applications of deep learning in food volume estimation that relied on relative estimations, this provides an avenue for more absolute volume calculations to be performed.

Myers et al. trained a CNN-based system with multiple RGB-D images taken of both real food and plastic food models to recognize and predict depth maps [110]. When 2D RGB photos of fully plated meals were tested with the system, it was able to generate a voxel depth map based on the visible food surfaces. Though the researchers were not able to achieve optimal accuracy with some test foods, some of which encountered absolute volume errors of up to 600ml, the technology showed promising results and could be a suitable way to extrapolate data from occluded sections of food images. 

Christ et al. developed a system that worked on similar principles, but instead of deriving absolute food volume, the system was designed to assist diabetics with estimating servings of carbohydrates, referred to in the paper as bread units (BU) [120]. This was done by training the system with a set of RGB-D images that had been annotated with the corresponding number of contained BUs by human experts. Depth maps were able to be accurately predicted from 2D RGB images fed into the system, achieving an average error of only 5% [120]. 

**Figure 5:**
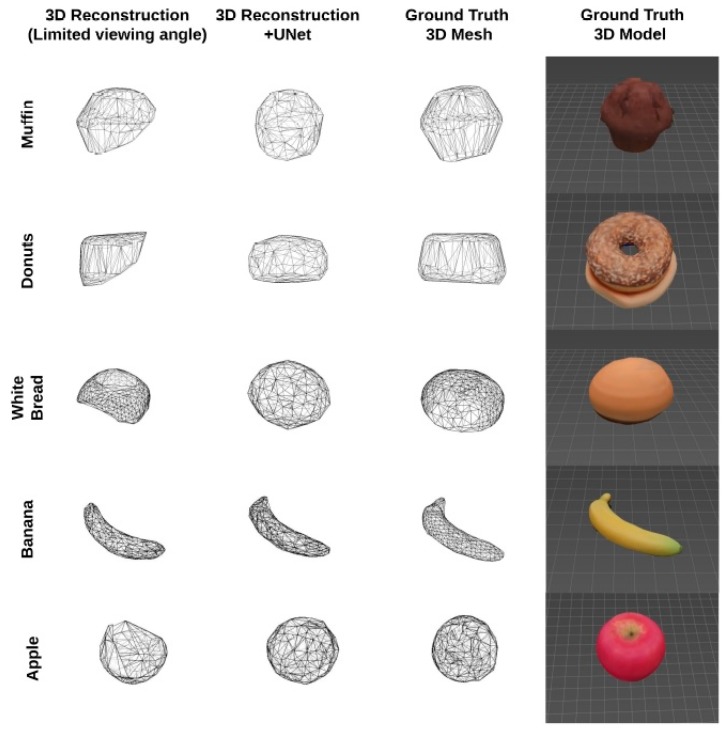
3D reconstruction of various food models with deep learning view synthesis, Lo et al. 2019.

More recently, Lo et al. trained a separate system with an extensive 3D food model database that consisted of 20,000 pairs of depth images per type of food [108]. These images were taken from opposing views and allowed the system to be familiar with occluded angles. When new single-view images were supplied to the system, the system was able to synthesize the occluded side in 3D space based on the training information, therefore allowing the full 3D mesh and volume to be determined (Figure 5) [109]. In two separate studies, researchers were able to determine food volume with promising results, showing an average accuracy of 98% and 92% respectively [108,109]. However, in free-living conditions, foods of a similar category may vary in shape, and can be difficult to predict unless a 3D-model food database of sufficient size and complexity is developed. 

Despite the advances in deep learning and computer vision technologies, the practicality of the approaches must also be considered. To be able to effectively utilize the application of deep learning, the methods discussed above require considerably larger datasets, ranging from 100 to 20,000 images per individual food type [108,109,110,120]. As such, the development of datasets will be exponentially tedious with the increasing number of food types and cuisines considered. A recent review by Zhou explored a few key limitations on the current applications of deep learning in food [115]. For the technology to be applicable for general public use, thousands of foods will need to be photographed, annotated and consolidated into a central database. Tapping into openly available resources such as the internet, social media and volunteers for data collection may be an appealing solution, but researchers will run the inevitable risk of collecting inconsistent and inaccurately labelled data which may further complicate the process. The use of extremely large databases will also require significant storage space and processing power, potentially requiring the use of cloud resources and remote processing, restricting their use in an offline setting. These barriers will need to be overcome if deep learning is to be used as part of the volume estimation process. 

### 5.3. Database Dependency

As with current dietary assessment practices, the accuracy of the database used is quintessential in ensuring the precision of the final derived result [20,32]. This could be problematic in the Southeast Asian region. Given the vast array of different local foods and delicacies, different countries have their own independently managed nutrient databases, and there is limited comparability between countries [121,122,123,124]. There are variations in the methods used to evaluate nutrients, and some databases may not include the full range of nutrients [122,124]. Variations in the agricultural origin, processing steps, as well as recipes of available foods can also contribute to significant disparities in nutrient values [121,124]. 

Density is an important factor in image-based assessment that needs to be considered in the conversion of food volume to calories [125,126]. Most large-scale nutrition databases do not include density as a factor in their entries, instead relying on mass as a comparative unit of measurement [125,126]. The common way of converting weight to volume involves the use of standard references such as typical sizes of fruit or household measures such as cups or tablespoons. However, research has proven this method to be largely inaccurate with significant differences found in 80% of tested foods [125,127]. At present, the most extensive food density dataset is maintained by FAO, but many entries are single-ingredient foods as opposed to composite meals that would be common in real-world scenarios [128]. Furthermore, there is limited representation of Asian foods, which makes the translation of such information to the Southeast Asian databases difficult.

Even if density factors are fully established, there may be limitations with nutrient determination due to the differences between absolute and bulk density [126]. Xu et al. commented on the difficulty of measuring food items such as salad leaves given that their calculable volume from a single-angle image will not take into account the air pockets and spaces between the leaves [129]. Breads that are baked with longer proofing times can also be less dense than their less-leavened counterparts; sauces and stews that are reduced or have syrups added may be of a much thicker consistency, and therefore denser than water or oil of the same volume. These discrepancies in volume measurements can lead to significant inaccuracies when used in deriving nutrient calculations.

For digital food volume measurement techniques to be successful, current databases will need to be evaluated and improved upon to ensure that accurate and appropriate information can be calculated [20].

## 6. Application to the Southeast Asian Consumer

Given the increasing adoption of internet and smartphone use in the Southeast Asian region, these personal devices may appear to be a favorable way to interface with users. To make the technology as ergonomic as possible in order to improve compliance rates, the number of steps and burdens on the respondents should be minimized. Applications that require users to carry around additional accessories or fiducial markers as part of the data collection process may also be less favorable to users. Applications should also limit the amount of required user inputs so as to make the process less frustrating for users. It should also be a tool that allows data to be derived from a single food photo. An ideal point to work towards would be to allow users to take a shot of their food from any particular angle without any additional restrictive criteria.

Though the use of personal smartphone devices could be a convenient means of delivery for some, the low SES population groups that are most vulnerable to the impact of poor nutrition may not be able to afford the most advanced technologies. If food volume estimation is to be integrated into current digital applications for effective public health use, developers will need to be wary that their requirements do not outstrip the technological capacity of the average consumer device. Careful selection of the appropriate volume estimation technologies is also crucial as auxiliary systems such as image and nutrient databases will have to be developed for applications to be successful, and the use of advanced methods may limit the usability of the system. 

The act of capturing a photo of all consumed meals may be a challenge for Southeast Asian consumers. Communal meals or banquets may make the act of recording dietary intake difficult [44]. Foods that one chooses to consume are generally picked out from the sharing platter just before eating. Additionally, piling a variety of food on one’s own plate is considered rude in many social settings. Foods at some Chinese banquets that are brought out sequentially throughout the course of the meal may require individuals to take multiple photos throughout the course of the meal. 

Certain types of foods may be difficult to differentiate if they are based solely on a single image. Foods such as curries or soups may be opaque, and when served in bowl, could obscure many details within the dish. Sauces that are poured over rice and breads may also be soaked up and become difficult to quantify. Furthermore, soups, sauces and curries can have a large variance in nutritional value depending on the type of ingredients used, even if they appear to be visually similar. In a similar strand, beverages such as sugar-free cola drinks will be indistinguishable from their regular varieties in an image. Many of the breads or wraps common in Southeast Asia can also have a range of different fillings which will significantly affect the nutritional content of the food. To improve accuracy and reduce the likelihood of incorrect food identification, these foods may require additional input from users. Apps could provide lists of suggested prompts to identify more details for users to select from: such as the type of filling contained in a sandwich; if a sauce or curry contains coconut milk; or a particular beverage presented in an image is sugar-free.

Dishes like curries and braised foods can have their sauce and solid items served in different proportions. In nutrient databases, solid components and the accompanying sauces are typically categorized together as a composite dish. However, separate nutrient values are not provided for sauces and the solid ingredients. This will lead to inaccuracies when converting calculated food volume into calories. Future technologies may need to explore solutions to identify and separate liquid and solid components of a single food item. 

Measuring food intake from food photographs can also be deceiving. Some components of meals are sometimes not fully consumed, in the case of soups and gravies which can often be left unfinished. Inedible parts of the meal such as animal bones, nut and clam shells or certain herbs and spices may be present in the photograph but are duly discarded. This could contribute to inaccurate approximations of consumed volume. In the context of shared meals, taking pictures of sharing platters could also portray an inaccurate estimation of the amount consumed by the individual. This requires individuals to be more aware of only capturing food volume that they are consuming. 

Given the large degree of dietary variation within Southeast Asia, there is a vast array of different dishes and ingredients that need to be collated. Databases will then be required to be very extensive to capture information accurately. Language barriers may also make it difficult to apply a single tool across the region. Therefore, applications and databases will need to be localized for them to work effectively with different populations.

## 7. Conclusions and Recommendations

This paper has explored the cultural hurdles to dietary assessment in the Southeast Asian region, the limitations of the current practices and approaches, and has reviewed novel ideas and concepts that aim to improve on these limitations. New computer vision technologies, especially smartphone-based tools incorporating machine learning and depth sensing, have considerable potential for nutritional precision on an individual level, as well as scalability to the larger population. Smartphone penetration in the Southeast Asian region has increased rapidly in recent years and the region may prove to be a fertile test bed for the development and integration of such applications. It is an advantageous time to capitalize on these advancements, and the development of a robust automated digital dietary assessment tool will likely enable governments, healthcare institutions, researchers and dietitians to be able to gather effective nutritional insight and combat the rising rates of obesity and diabetes in the region.

That being said, we recognize certain limitations in this review. Firstly, this paper does not fully explore the logistical and regulatory requirements of such a task, and more research will be required to determine the practical feasibility of applying such a technology in the field. The creation of a homogenized nutritional dataset with the inclusion of density factors to support such an endeavor is likely to be a challenging and expensive task, and will require much consideration. Secondly, given the rising popularity of food photography and widespread use of smartphones, this review has also focused primarily on image-based methods of digital dietary assessment and does not consider other forms of digital methods such as bar code scanners or text-based recording in the discussion. Lastly, though this review attempted to provide an extensive discussion about the complexity of the Southeast Asian diet, it is by no means comprehensive and we recognize that there are many ethnic and regional intricacies that were not fully articulated. As such, we recommend that future researchers intending to implement the technology carefully consider eating behaviors and practices unique to the localized environment in the design of their application.

The central axis of nutrition is the precise estimation of an individual’s food intake. Food intake measurements are critical for both prescriptive and diagnostic approaches in the provision of an optimal diet. Despite many decades of work in this area, there still remains considerable limitations. With the advent of the digital revolution and the use of machine learning and artificial intelligence, we now have the potential to greatly improve our ability to estimate food intake on both an individual and on a population level. Indeed, the time has come to recognize the limitations of the conventional methods of estimating nutrient intake and embrace the current advances in computing, technology and machine learning to resolve one of the most important questions in human nutrition.

## Supplementary Materials

The following are available online at https://www.mdpi.com/2072-6643/12/4/1167/s1, Table S1: Table of image-based food volume estimation methods.
