# Supplementary material for: Current Developments in Digital Quantitative Volume Estimation for the Optimisation of Dietary Assessment
Source: Nutrients. 2020 Apr 22;12(4):1167. doi: 10.3390/nu12041167 (PMC7231293; doi:10.3390/nu12041167)
Supplement: Supplementary file 1 [file nutrients-12-01167-s001.pdf]

Authors	Input Type	Image Capture Device	Scale Measurement	Volume Measurement Principle	No. Foods Tested	Type of Foods Tested	Environment	Relative Errors
Woo I, Otsmo K, Kim Sy, Ebert DS, Delp EJ, Boushey CJ [75]	Single RGB Image	Smart Phone	Physical<br><br>Reference Card	Geometrical mapping (spherical and prismatic)	7	Single foods - Western	Controlled	5.65% (Spherical)<br><br>28.85% (Prismatic)
Yue YF, Jia WY, Sun M [85]	Single RGB Image	Smart Phone	Physical<br><br>Standard tableware size	Pixel Density - Calculations derived from coordinates	6	Single foods - Replicas	Controlled	3.41%
Jia WY, Yue YF, Fernstrom JD, Yao N, Sclabassi RJ, Fernstrom MH, Sun M [86]	Single RGB Image	Camera with LED Spotlight	Physical or Digital<br><br>Circular reference card or projected reference template	Geometric Modelling<br><br>Prism / Cylinder / Cone / Sphere	6	Single foods - Replicas	Controlled	12.01% (Plate Method)<br><br>29.01% (LED Method)
Fang SB, Shao ZM, Kerr DA, Boushey CJ, Zhu FQ [87]	Single RGB Image	Food images collected via mobile food record (mFR) app	Physical<br><br>Reference Card	Deep Learning<br><br>Predictive models based on heatmaps corresponding to food-energy distribution within training images	79	Segmented single and mixed foods	Controlled	38.20%
Dehais J, Anthimopoulos M, Shevchik S, Mougiakakou S [88]	Stereo RGB Images	Food images captured with smartphone cameras	Physical<br><br>Reference Card	Depth Mapping - 3D reconstruction via stereo imaging.	77	Segmented mixed and single foods	Controlled	7.4 - 9.8% (MAPE)
Chae JH, Woo Ii, Kim SY, Maciejewski R, Zhu FG, Delp EJ, Boushey CJ, Ebert DS [89]	Single RGB Image	Smart Phone	Physical<br><br>Reference Card	Geometric Modelling - Cylinders and Flat-top Solid Shapes	26	Single foods	Controlled	11.1% (Cylinders)<br><br>11.7% (Flat-top Solid)
Xu C, He Y, Khanna N, Boushey CJ, Delp EJ [90]	Single RGB Image	Smart Phone	Physical<br><br>Reference Card	Deep Learning - 3D reconstruction based on detected poses	5	Single foods	Controlled	7.22%
He Y, Xu C, Khanna N, Boushey CJ, Delp EJ [91]	Single RGB Image	Smart phone	Physical<br><br>Reference Card	Deep Learning - Area-based weight estimation	1	Single food	Controlled	10%
Rahman H, Li Q, Pickering MR, Frater M, Kerr D, Bouchey CJ, Delp EJ [92]	Stereo RGB Images	Smart Phone	Physical<br><br>Reference Card	Depth Mapping - 3D point cloud generation from stereo matching	6	Whole Fruits	Controlled	7.70%
Puri M, Zhu ZW, Yu Q, Divakaran A, Sawhney H [93]	3 RGB Images	Smart phone	Physical<br><br>Reference Card	Depth Mapping	6 sets of food (20 total items)	Segmented mixed foods - Western	Controlled	4.69%
Martin CK, Kaya S, Gunturk BK [94]	Single RGB Image	Smart Phone	Physical<br><br>Reference Card	Pixel Density - Area to volume calculations	Nil	Single foods - Western	Controlled	Nil<br><br>No results presented
Rhyner D, Loher H, Dehais J, Anthimopoulos M, Shevchik S, Botwey RH, Duke D, Stettler C, Diem P, Mougiakakou S [95]	Stereo RGB Images - (Top and 15 deg from midline)	Smart Phone	Physical<br><br>Reference Card	Depth Mapping	114 Hospital Meals	Segmented composite meals - Western	Controlled	26.2%<br><br>55.9% improved accuracy (app vs no app)
Ege T, Shimoda W, Yanai K [96]	Single RGB Image	Any	Physical - Rice grain	Pixel Density<br><br>Food category dependent quadratic equations	100 Categories	Composite meals - Japanese	Free Living	5.55%
Akpa EAH, Suwa H, Arakawa Y, Yaumoto K [97]	Single RGB Photo	Smartphone	Physical<br><br>Cutlery (Chopsticks)	Pixel Density<br><br>Algorithmic calculations based on derived height and diameter	15	Composite meals - Japanese bowl foods	Controlled	6.65%
Liang YC, Li JH [98]	Stereo RGB Images (Top and Side)	Smart Phone	Physical<br><br>Coin	Pixel density<br><br>3 Formulas used depending on object shape (ellipsoidal / irregular / columnar)	19	Single foods	Controlled	12.40%
Villalobos G, Almaghrabi R, Puladzadeh P, Shirmohammadi S [99]	Stereo RGB Images	Smart Phone	Physical<br><br>User's thumb or coin	Pixel Density	Nil	Nil	Nil	Nil
Pouladzadeh P, Shirmohammadi S [100]	Stereo RGB Images	Smart phone	Physical<br><br>User's thumb	Pixel Density	5	Single foods - Western	Controlled	14%
Subhi MA, Ali SH, Ismail AG, Othma M [101]	Stereo RGB Images	Stereoscopic Eyewear	Digital<br><br>Stereoscopic Cameras	Geometric Modelling<br><br>3D boundary boxes derived from identified object features	4	Single foods	Controlled	8.50%
Shang JQ, Duong M, Pepin E, Zhang X, Sandara-Rajan K, Mamishev A, Kristal A [102]	Revolving Video	Smart Phone attached with SLS device	Digital<br><br>Structured Light	Depth Mapping	Nil	Nil	Controlled	Nil<br><br>No results presented
Makhsous S, Mohammed HM, Schenk JM, Mamishev AV, Kristal AR [103]	Revolving Video	Smartphone	Digital<br><br>Projected Structured Light System	Depth Mapping	20	Single and mixed foods	Controlled	11.00%
Tanno R, Ege T, Yanai K [104]	Single RGB Image	Any	Digital<br><br>Apple AR Toolkit	Geometric Modelling<br><br>Application of quadratic equations to identified boundary box	3	Single and composite foods	Controlled	12.26%
Yang YF, Jia WY, Bucher T, Zhang H, Sun MG [105]	Single RGB Image	Smart phone with augmented reality function	Digital<br><br>Augmented Reality	Geometric Modelling<br><br>Predefined AR-projected shapes	15 Food Replicas 14 Actual Foods	Single foods - Mixture of replica and real foods	Controlled	26.97%
Lo FPW, SunYN, Qiu JN, Lo B [107]	Single RGB-D Image	Smartphone with Depth Sensing Capabilities	Digital<br><br>Depth Sensing	Integrated Approach (Depth Mapping and Deep Learning)	8	Single foods	Controlled	6.88%
Zhu FQ, Bosch M, Woo I, Kim SY, Boushey CJ, Ebert DS	Single RGB Image	Smart Phone	Physical<br><br>Reference Card	Geometrical mapping (spherical and prismatic) to determine food volume	7 Foods	Single,, Western	Controlled	3.4% - 56.4%
Lo FPW, Sun YN, Qiu JN, Lo B [108]	Single RGB-D Image or Video	Smartphone with Depth Sensing Capabilities	Digital<br><br>Depth sensor	Depth Mapping<br><br>Dense 3D reconstruction based on training models	8	Single foods	Controlled	7.71%
Myers A, Johnston N, Rathod V, Korattikara A, Gorban A, Silberman N, Guadarrama S, Papandreou G, Huang J, Murphy K [109]	Single RGB Image	Smart Phone	Digital<br><br>CNN Predicted Depth	Depth Mapping - 3D meshing of CNN predicted depth parameters	42 Food Items arranged in 11 mealsets	Single foods - Replicas	Controlled	% not stated. Max volume discrepancy up to 600ml
Ando Y, Ege T, Cho J, Yanai K [110]	Single RGB-D Image	iPhone X	Digital<br><br>Depth-sensing stereoscopic cameras	Pixel Density<br><br>Deph is derived from surface of food to reference plane	3	Japanese Foods	Controlled	5.13%
Zhang WY, Yu Q, Siddiquie B, Divakaran A, Sawhney H [111]	Single RGB Image	Smart Phone	Nil	Pixel density<br><br>Depth images provide better estimates of portion size	15	Single foods - Western	Controlled	Nil<br><br>No results presented
Okamoto K, Yanai K [112]	Single RGB Image	Any	Physical<br><br>User's belongings (Wallet / Card / Smartphone case)	Pixel Density<br><br>Visible food area converted into volume via quadratic equations	20	Single foods - Japanese	Controlled	21.30%
Jia WY, Chen HC, Yue YF, Li ZX, Fernstrom J, Bai YC, Li CL, Sun MG [113]	Multiple RGB Images	eButton	Physical<br><br>Tableware size	Geometric Modelling - Computer-assisted model matching with identified features	100	Single and Mixed - Western & Asian	Controlled	2.80%
Ege T, Yanai K [115]	Single RGB Image	Any	Nil	Deep Learning<br><br>Matching calorie-annotated food images with the target foods	15 Categories	Composite meals - Japanese	Free Living	27.93%
Isaksen R, Knudsen EB, Walde AI [116]	Single RGB Image	Any	Nil	Deep Learning<br><br>Weight-annotated food training images	8	Single foods	Controlled	8.95%
Chokr M, Elbassuoni S [117]	Single RGB Image	Any	Digital<br><br>Deep Learning	Deep Learning<br><br>Individual food sizes tagged to training images	6	Single foods (Fast Food)	Controlled	0.0933 kcal (mean absolute error)
Christ PF, Schlecht S, Ettlinger F, Grun F, Heinle C, Tatavatry S, Ahmadi SA, Diepold K, Menze BH [119]	Single RGB Image or Single RGB-D Image	Smartphone	Digital<br><br>Depth prediction if depth information not provided	Deep Learning<br><br>Depth and carbohydrate prediction based on training datasets	60	Single and composite foods - Western	Controlled	12.90%
Al-Maghrabi R [128]	Single RGB Photo	Smartphone	Physical<br><br>User's thumb	Pixel Density<br><br>Algorithmic calculations based on calculated pixel area	32	Segmented single and mixed foods - Western	Controlled	6.84%
Xu C, He Y, Parra A, Delp EJ, Khanna N, Boushey CJ [129]	Single or Multiple RGB Images	DSLR / Smart Phone	Physical<br><br>Reference Card	Deep learning - Single View 3D reconstruction with prior training<br><br>Depth Mapping - Multi-view derivation from silhoettes	19	Single and Mixed Foods	Controlled	17.86% (Single-view)<br><br>10.13% (Multi-view)
